# Attributional style and depressive symptoms in a male prison sample

**DOI:** 10.1371/journal.pone.0190394

**Published:** 2018-02-14

**Authors:** Danny J. O’Sullivan, Maura E. O’Sullivan, Brendan D. O’Connell, Ken O’Reilly, Kiran M. Sarma

**Affiliations:** 1 Department of Psychology, National Forensic Mental Health Service, Dundrum, Dublin, Ireland; 2 Department of Psychology, Irish Prison Service, Longford, Ireland; 3 Department of Psychiatry, Trinity College Dublin, Dublin, Ireland; 4 School of Psychology, National University of Ireland Galway, Galway, Ireland; Xi'an Jiaotong University School of Medicine, CHINA

## Abstract

The reformulated learned helplessness model proposes that people who tend to make internal, stable, and global attributions in response to uncontrollable aversive events are more likely to develop depression. The present study sought to investigate the nature of the relationship between attributional style and depression in a male prison sample. One hundred and one adult male prisoners from four medium security prisons in Ireland completed the Attributional Style Questionnaire and measures of depression (BDI-II) and anxiety (BAI). Severity of self-reported depressive symptoms in the present sample was comparable to other prison and clinical samples, but higher than community samples. Participants were more severely affected by depressive symptoms than anxiety. The original attributional dimensions (i.e. internal, stable, and global) predicted a significant amount of variance in depression, but the model was not significant after controlling for anxiety. A subsequent regression model, comprising attributional dimensions for both negative events and positive events including a measure of ‘uncontrollability’, accounted for 35% of the variance in depression and the model retained significance while controlling for anxiety. An attributional model of depression may be relevant to the prison population and could provide a valid insight into the development and treatment of depressive symptoms in prisoners. The findings are interpreted in relation to previous research and implications for theory, clinical practice, and rehabilitation are discussed.

## Introduction

The prevalence and severity of mental health problems is higher in prison populations than in community populations [1], [2]. A systematic review involving 28,361 male prisoners reported rates of 10.2% for depression and 3.6% for psychosis [3]. In Ireland prevalence rates of 6.3% for major depressive disorders, 5.4% for anxiety disorders, and 3.9% for psychotic disorders were reported in a male prison sample [4]. The severity of self-reported symptoms associated with mental health problems have also been examined in prison populations. In one study [5] 8.1% of prisoners reported severe levels of depressive symptoms on the BDI-II, and 2.3% reported severe levels of anxiety symptoms on the BAI. Elsewhere severe depressive symptoms [6] and high levels of hopelessness have been found amongst the general prison population [7]. The prevalence of mental health disorders and severity of mental health symptoms in prisoners are indicative of the vulnerability of this population.

### Psychological vulnerability in the prison population

Several aspects of imprisonment can impact on the mental health of prisoners. These include deprivation of liberty, overcrowding, hygiene and sanitation, inadequate healthcare, aggression and violence, solitude, and the availability of psychoactive substances [8]. The research indicates that a significant minority of prisoners experience mental health difficulties, suggesting that some prisoners are more vulnerable than others. There is evidence that individual cognitive differences, including attributional style, moderate the impact of imprisonment on psychological well-being and adjustment [9–11]. This diathesis-stress approach emphasizes how underlying cognitive vulnerabilities predispose an individual to psychological distress in the context of a stressful life event, and provides a model for understanding how individuals differ in their response to imprisonment.

### Learned helplessness and imprisonment

The reformulated learned helplessness model (RLHM) [12] is based on a diathesis-stress interaction. It proposes that the symptoms of depression can be triggered by uncontrollable aversive circumstances, moderated by a cognitive vulnerability. This cognitive vulnerability, namely depressogenic attributional style, is a relatively stable tendency to explain negative events in terms of internal (e.g. “it’s due to me”), stable (e.g. “it will be there in the future”), and global causes (e.g. “it affects everything I do”).

Schill and Marcus [13] have argued that imprisonment involves prolonged exposure to uncontrollable aversive circumstances that can lead to a state of learned helplessness, yet there are surprisingly few studies on the association between attributional style and depression in prisoners. One such study described by Peterson and Seligman [9] found that significant increases in depressive symptoms after a period of imprisonment were strongly associated with depressogenic attributional style at the beginning of imprisonment. So individuals who tended to attribute negative events to internal, stable, and global causes were more likely to experience increased depressive symptoms in response to imprisonment. These findings support the model that depressogenic attributional style is a characteristic vulnerability, which in response to an environmental stressor increases the risk for depression.

### Attributional style and psychopathology

The association between attributional style and depressive symptoms [14–16], anxiety symptoms [17], [18], hopelessness [19], suicidal ideation [20], [21], and PTSD [22] is supported in the literature. In a forensic context, attributional style has been implicated in the associations of oppositionality and delinquency with depressed mood [23], and in desistance from crime [24]. These studies illustrate the important role attributional style plays in psychological well-being and the potentially important role attributional style could play in understanding criminal behavior and reform.

### Building the present study

There are a number of variables that affect the relationship between attributional style and depressive symptoms. For example, it has been reported that anxiety is relevant [25], which is not surprising given that anxiety frequently co-occurs with depression [26]. But RLHM is specific to depression rather than general psychopathology. In addition, the concept of ‘perceived uncontrollability’ was central to the development of depression in RLHM, yet this important variable has largely been neglected [27]. ‘Attributions of uncontrollability’ (i.e. the extent to which the causes of events are perceived as uncontrollable) has been shown to increase the likelihood that a person with a depressogenic attributional style will develop depressive symptoms in response to an adverse event [28]. The distinction between attributions for positive events versus negative events (i.e. event valence) is relevant, because individuals who adopt an enhancing attributional style for positive events (i.e. attribute positive events to internal, stable, and global causes) experience lower levels of depression and hopelessness [29], [30].The role of individual variables that affect the relationship between attributional style and depression has been explored in these studies, but there is little evidence of any attempt to build a predictive model comprising the most salient of these individual variables in a clinically relevant sample. A model of the relationship between attributional style and depression may offer a new perspective on psychological vulnerability in prisoners, and may inform new methods of enhancing their psychological well-being. We expected that:

1. A model comprising the three original attributional dimensions (i.e. internal, stable, and global attributions) for negative events would account for significant variance in depressive symptoms as proposed by RLHM.
2. The model would retain significance after controlling for the variance explained by anxiety. This hypothesis sought to test the specificity of the original attributional model to depression in this sample.
3. Finally we sought to build a parsimonious attributional model by including the fewest necessary variables to predict the largest proportion of variance in depression. We expected that the parsimonious model would comprise attributional dimensions for negative events and positive events, that attributions of ‘uncontrollability’ would contribute to this model, and that the model would account for significant variance in depression after controlling for anxiety.

## Method

### Design

A cross sectional design was employed. The predictor variables were internality, stability, globality, and uncontrollability (i.e. the dimensions of attributional style), and the criterion was depressive symptomatology. Sample size estimates using G*Power 3.1 [31] indicated that the present study required 85 participants, based on the required significance level (α = .05), the desired statistical power (1 – β = 0.8), the estimated effect size in the population (f^2^ = 0.15), and the number of predictors in the model (*m* = 4).

### Participants

Participants were sampled from four of the eight medium security prisons in the Republic of Ireland [32]. In total 101 participants were recruited. The sample ranged in age from 19 to 78 years, with a mean age of 32.9 years (SD = 12.4, mode = 27). Ninety-one participants described their nationality as Irish, five were from the United Kingdom, and the remaining five were from other EU Member States, Africa, and Asia. At the time of sampling 86 participants were serving a sentence, eight were on remand or awaiting trial, and four were concurrently serving a sentence and on remand. The average number of months since admission to prison for the sample was 30.1 (SD = 37.26), with most participants arriving to prison within the previous thirteen months. Participants were serving prison sentences for Drug Offences (n = 23), Murder (n = 17), Sexual Offences (n = 15), and Other Offences Against the Person (n = 12). The average number of years participants spent in formal education was 11.1 (SD = 3.35). Sixty participants reported having previously attended mental health services, either in prison or in the community. To ensure that the sample was representative of the medium security prison population in Ireland, participants were recruited from prisons across different counties and using a stratified random sampling method.

### Materials and measures

#### Attributional Style Questionnaire (ASQ)

The ASQ [33] is a self-report instrument that assesses causal attributions for six positive and six negative hypothetical events. Respondents are required to “write down the one major cause” for each of the twelve events and rate, on a seven-point scale, the extent to which they attribute the cause to internal versus external (e.g. 7 = totally due to me, 1 = totally due to other people or circumstances), stable versus transient (e.g. 7 = will always be present, 1 = will never again be present), and global versus specific factors (7 = influences all situations in my life, 1 = influences just this particular situation). The present study also included a fourth ‘uncontrollability’ dimension (e.g. 7 = I cannot control the cause of the situation, 1 = I can fully control the cause) [28].The psychometric properties of the ASQ have been investigated extensively. Studies reporting on internal consistency [16], test-retest reliability [32], analysis of factor structure [34], and analysis of concurrent and predictive validity [35] indicate that the ASQ can provide a reliable and valid measure of attributional style as a cognitive vulnerability to depression. The internal consistency of the ASQ in the present sample was acceptable (α = .74).

#### Beck Depression Inventory, Second Edition (BDI-II)

The BDI-II [36] is a 21-item self-report instrument for measuring the presence and severity of depressive symptoms. The items are consistent with diagnostic criteria for depressive disorders in the DSM-IV [37]. Items are rated on a four-point scale ranging from 0 to 3, reflecting increasing severity of each symptom. Total scores may be categorised as minimal (0–13), mild (14–19), moderate (20–28), or severe (29–63). The BDI-II is a reliable and valid psychometric instrument for measuring depression in clinical, non-clinical, and offender samples [38], [39]. The internal consistency of the BDI-II in the present sample was excellent (α = .92).

#### Beck Anxiety Inventory (BAI)

The BAI [40] is a 21-item instrument for measuring the presence and severity of self-reported anxiety symptoms. Respondents indicate how much they have been bothered by each symptom in the past week on a four-point scale ranging from ‘Not at all’ to ‘Severely’. Total scores may be categorised as minimal (0–7), mild (8–15), moderate (16–25), or severe (26–63). The BAI shows good internal consistency and adequate test-retest reliability [41]. Studies demonstrating the convergent and discriminant validity of the BAI have also been reported [42]. The internal consistency of the BAI in the present sample was excellent (α = .95).

#### Demographic questionnaire

A demographic questionnaire with 16 items was developed to obtain relevant information about participants’ current imprisonment status, and their educational and mental health histories.

### Procedure

Ethical approval for the study and consent procedure was granted by the Irish Prison Service (IPS) and NUI Galway University Research Ethics Committees. A list of eligible participants was generated using the Irish Prison Service database (PRIS) in the four medium security prisons that participated. Potential participants were selected using a stratified random sampling method whereby every third prisoner on each list was invited to meet with the researcher to discuss taking part in the study [43]. A convenience sample (n = 26) was recruited in one prison due to security concerns and limited resources within the prison. Potential participants were guided through the Participant Information Sheet and Consent Form. Each was invited to decline to participate if he was not satisfied with the information, if he was not satisfied that he fully understood it, or if he simply wanted no further involvement. Translated questionnaires or interpreters were not provided. Of the 156 who were invited and available to take part, 101 consented and completed the measures. Fifty five individuals declined to take part. These figures represent a 64.7% rate of acceptance and are comparable to other studies in Irish prisons, in which approximately one third declined to participate [43], [44]. Participants completed the measures on an individual basis, or in small groups of up to four, in the presence of the researcher. Some decided to complete the measures elsewhere in their own time. Those with literacy difficulties were assisted. The purpose of assisting participants was to maximize inclusion and data quality, and to reduce the cognitive and literacy burden. However, providing assistance may also have increased the likelihood of social desirability in completing the measures [45]. Participants were debriefed upon completion and vulnerable or distressed participants were referred to IPS psychology or mental health services.

### Data analysis

The data set was initially screened for errors and the distribution of scores for each measure was analyzed. Invalid and incomplete questionnaires (n = 9) were omitted. Independent samples T-tests were used to compare depression scores in the present sample to other prison, psychiatric, and community samples. The dimensions of the ASQ were normally distributed, but the Beck inventories were positively skewed (i.e. scores were more likely to fall at the lower end of the scales). Kendall’s tau (τ), a non-parametric statistic for analysing correlations between variables with non-normal distributions, was therefore used to examine bivariate relationships between the Beck inventories and the other variables. Multiple linear regression equations were conducted to evaluate the hypotheses. None of the prerequisite assumptions concerning sample size, multicollinearity, or distribution of scores was violated, suggesting that the regression model was appropriate for the present sample and applicable to the population. Raw data is available in S1 Dataset.

## Results

### Depression and anxiety

The mean BDI-II score was 18.02 (SD = 12.24), falling within the ‘mild’ range of depressive symptoms. Depression scores in the present sample were comparable to other prison samples [7], [*t*(123) = 1.01, p = 0.314] and to psychiatric samples [36], [*t*(282) = 1.51, p = 0.132], but significantly higher than community samples [36], [*t*(152) = 4.26, p < .001]. The mean BAI score was 12.66 (SD = 13.66), which also falls in the ‘mild’ range. Analysis of the distribution of scores showed that the occurrence and intensity of depressive symptoms were more frequently endorsed than anxiety symptoms. For example, 37.6% of the sample reported moderate to severe levels of depressive symptoms, with 29.7% reporting moderate to severe levels of anxiety symptoms. This suggests that the sample was more severely affected by symptoms of depression than anxiety. The distribution of scores on the BDI-II and BAI is presented in Table 1.

**Table 1:** Frequency distributions for the qualitative categories on the beck inventories.

Measure	Minimal	Mild	Moderate	Severe
BDI-II	47 (46.5%)	14 (13.9%)	17 (16.8%)	21 (20.8%)
BAI	46 (45.5%)	24 (23.8%)	13 (12.9%)	17 (16.8%)

### Preliminary correlation analyses

Concerning the demographic and criterion variables, there was a significant negative correlation between age and anxiety (τ = -.211, p = .004), and between ‘Number of months since admission’ and anxiety (τ = -.153, p = .040). This suggests that the older the prisoner and the longer he was into his sentence the less likely he was to be bothered by anxiety symptoms. ‘Number of months to remission’ was not associated with depression or anxiety. A significant negative association was evident between ‘number of years in formal education’ and depression (τ = -.201, p = .006), and anxiety (τ = -.265, p < .001). Thus, participants spending less time in school were more likely to report increasing symptoms of depression and anxiety at the time of data collection. It is important to note however that causation cannot be inferred from any of these associations.

Unsurprisingly, a significant positive correlation was evident between depression and anxiety (τ = .550, p < .001). Concerning the predictor and criterion variables, depression showed significant positive correlations with internal (τ = .227, p = .002) and global (τ = .236, p = .001) attributions for negative events, and significant negative correlations with internal (τ = -.218, p = .003) and controllable (τ = -.150, p = .042) attributions for positive events. Similarly, anxiety showed significant positive correlations with internal (τ = .218, p = .003) and global (τ = .180, p = .015) attributions for negative events, and a significant negative correlation with controllable (τ = -.178, p = .016) attributions for positive events. The correlation matrix is in S1 Table.

### Hypothesis testing

The first multiple regression equation was conducted to examine whether a model comprising the three original attributional dimensions for negative events (i.e. internality, stability, and globality) would account for significant variance in depression scores on the BDI-II. In this case all of the predictor variables were entered into the model simultaneously. The model explained 19.3% of the variance in depression (R^2^ = .193), which was statistically significant [F (3, 86) = 6.89, p < .001]. ‘Globality’ (β = .32, p = .008) and ‘internality’ (β = .22, p = .044) each made a significant and unique contribution in the model, however ‘stability’ (β = -.05, p = .679) did not. The first hypothesis was supported in relation to ‘internality’ and ‘globality’.

In the second equation, it was expected that the original attributional model for negative events would account for significant variance in depression above that shared with anxiety. To control for anxiety, total BAI scores were entered in the first block of a hierarchical multiple regression equation. This showed that anxiety made a significant contribution to explaining variance in depression scores [R^2^ = .50, F (1, 88) = 88.49, p < .001]. The addition of the attributional variables in the second stage corresponded with a non-significant increase in variance (R^2^ change = .04, p = .089). None of the attributional variables made a significant unique contribution to explaining additional variance in depression above that already explained by anxiety. This suggests that the original attributional model as proposed by RLHM is not specific to depression in this sample and may perhaps better predict levels of general psychopathology in this population (e.g. comorbid anxiety and depressive symptoms). The null hypothesis was retained.

Finally, the present study sought to build a parsimonious predictive model of depression. A preliminary regression analysis was conducted in which all theoretically derived predictor variables were entered into the model to investigate which best predicted the criterion [46]. Using this method, four of the possible eight attributional dimensions were identified as potentially important predictors for the parsimonious model. These variables were internality and uncontrollability for negative events, and internality and globality for positive events. The regression was rerun using these four variables as predictors. Again the model significantly predicted the criterion, accounting for 35% of the variance in depression [R^2^ = .35, F (4, 85) = 11.42, p < .001]. In addition, each of the predictors made a significant unique contribution. Internality for positive events made the strongest unique contribution (β = -.44, p = .006), followed by internality for negative events (β = .34, p = .007), uncontrollability for negative events (β = .24, p = .009), and globality for positive events (β = .23, p = .012). Another hierarchical multiple regression equation was conducted to investigate whether the model could account for variance specific to depression beyond that shared with anxiety and ‘number of years in formal education’. Because the latter was negatively correlated with depression, it was entered into the regression equation to control for potentially overlapping variance with the predictors. No other demographic variable warranted inclusion on this basis. The findings of this equation are presented in Table 2.

**Table 2:** Results of the hierarchical regression attributional model of depression.

*Predictor*	*B*	*St*. *Error B*	β	*Sig*	*R*^*2*^
Block 1					
Constant	27.335	4.659			
Years in Education	-.840	.402	-.230	.040	.053
Block 2					
Constant	9.527	4.019			
Years in Education	.018	.311	.005	.955	
Anxiety	.655	.079	.710	.000	.501
Block 3					
Constant	6.733	8.011			
Years Education	-.048	.287	-.013	.869	
Anxiety	.513	.079	.555	.000	
Internality Positive	-3.544	1.052	-.272	.001	
Internality Negative	2.318	.835	.218	.007	
Uncontrollability Negative	1.575	.717	.167	.031	
Globality Positive	1.644	.942	.138	.085	.607

As illustrated in Table 2, ‘years in education’ was entered in block 1 of the hierarchy. In this first stage a significant amount of variance in the criterion was predicted [R^2^ = .05, F (1, 78) = 4.36, p = .040]. The addition of anxiety in block 2 significantly increased the variance already explained by ‘years in education’ [R^2^ change = .45, F (2, 77) = 38.72, p < .001]. The attributional variables were added in block 3 and made a significant contribution to the model after controlling for ‘years in education’ and anxiety [R^2^ change = .11, F (6, 73) = 18.82, p < .001]. Internality for positive events (β = -.27, p = .001), internality for negative events (β = .22, p = .007), and uncontrollability for negative events (β = .17, p = .031) each made a significant unique contribution. Globality for positive events did not (β = .14, p = .085).

## Discussion

This study sought to investigate the relationship between attributional style and depressive symptoms in a male prison sample. Moderate to severe levels of depression and anxiety symptoms were reported by a substantial proportion of this sample. Our findings support the argument that, although affective difficulties are not universal amongst prisoners, this population appears to better resemble psychiatric outpatients than the general population in terms of self-reported depressive symptoms [5]. In addition, the finding that more participants in the present study reported moderate to severe levels of depression than anxiety appears to be consistent with research suggesting that, other than substance use disorder, depression represents the most significant mental health problem facing prisoners [47], [48].

Regarding our predictions, we sought to test whether the original reformulated learned helplessness model (RLHM) was related to depression, and whether this model was specific to depression. The initial regression model comprising the three original attributional dimensions for negative events showed that both internality and globality, but not stability, made a significant unique contribution to explaining the variance in depression. This model did not however make a unique contribution to depression beyond the variance explained by anxiety. While not entirely consistent with RLHM, our finding was consistent with research showing that depressogenic attributional style for negative events represents a general vulnerability for both depression and anxiety [25], [49]. This is not surprising given the high comorbidity rates often observed between depression and anxiety [26].

A subsequent parsimonious regression model of depression, comprising attributional dimensions for negative events (internality and uncontrollability) and positive events (internality and globality), explained additional variance in depression compared to the original model, and made a significant and unique contribution to depression when controlling for anxiety. Thus, participants who tended to attribute the causes of negative events to internal and uncontrollable factors (e.g. “It’s me and I can’t do anything about it”) and who tended to attribute the causes of positive events to external and specific factors (e.g. “It’s not due to me and it only affects this situation”) were more likely to report higher depression scores, independent of anxiety. Our findings suggest that depressed prisoners engage in unhelpful attributions perpetuating self-blame and helplessness when things go wrong, and tend to regard positive events as isolated incidents involving little or no personal contribution.

### Theoretical implications

Our finding that attributional style, when considered in the context of both positive and negative events, can provide a model that is specific to depression is supported in the literature [50]. This has implications for RLHM which has focused primarily on the role of attributional style in the context of negative events. Exploring the relationship and interaction between attributional style for positive and negative events, which are not simply inversely related, may enhance the attributional account that is specific to depression, and not related to other psychopathologies such as anxiety. Evidence from the literature and the present study suggest that pursuing further this line of research could prove progressive from a theoretical point of view. The same might also be said for the dimension of uncontrollability.

Regarding uncontrollability, it has been reported elsewhere that prisoners with greater perceptions and expectations of control experience less distress and adjust better to imprisonment [51]. It is not surprising therefore, that perceived uncontrollability for negative events was an important attributional variable in predicting depression in the present study. Despite its centrality to the original theory, the uncontrollability dimension has not been extensively researched. The findings herein support its relevance to an attributional model of depression in a prison sample and warrant its inclusion in future measures of attributional style.

### Clinical implications

Modifying a depressogenic attributional style could be an effective therapeutic strategy to restoring hopefulness and increasing resilience [19]. Several studies have reported that attributing positive events to internal, stable, and global causes is associated with recovery from depression and lower risk for relapse [29], [30]. These findings suggest that it could be therapeutically beneficial to provide vulnerable prisoners with a skill-set that helps them modify their depressogenic attributions, specifically targeting internal and uncontrollable attributions for negative events and internal and global attributions for positive events. The particular relevance of uncontrollable attributions to the present sample is supported by evidence suggesting that increasing the perceived controllability of the environment in individuals who are incarcerated is associated with better adjustment and decreased levels of distress [51], [52]. ‘Self-Administered Optimism Training’ represents a promising and potentially cost-efficient approach to reducing the cognitive vulnerability of depressogenic attributional style [53]. It is a minimally supervised training program that teaches participants to develop more adaptive attributions for daily events. It has been associated with greater decreases in depressogenic attributional style and with decreases in depression [53]. With increasing financial constraints placed on State run institutions in recent years, self-administered optimism training could provide a cost effective, albeit incomprehensive, method for prison Psychology Services to help low priority prisoners presenting with cognitive vulnerabilities to depression, while devoting greater time to prisoners presenting with more acute psychological and mental health difficulties. Investigating the impact of self-administered optimism training on depressive symptoms in a prison sample represents a potentially valuable avenue for future research.

### Crime relevant implications

The reformulated learned helplessness model proposes that attributing negative events to external causes is adaptive to psychological well-being. However, Tennen and Affleck [54] (p41) have argued that the association between casual attributions and well-being may be too complex to conclude that “externalizing responsibility for bad events is universally adaptive”, particularly when it comes to serious and violent crimes. This point seems relevant in the context of promoting an enhancing attributional style in the prison population. In offenders, attributing the causes of criminality to stable and intentional factors has been associated with psychological distress, whereas attributions that engender expectations of change for the better and attributions that deemphasize global personality deficits have been associated with enhancing self-esteem and prosocial behavior [55]. Maruna [24] found that amongst offenders a depressogenic attributional style for negative events was negatively associated with desistance from criminal behavior, and an enhancing attributional style for positive events was positively associated with reform. It may be that controllability attributions play a role in desistance. For example, offenders are more likely to eventually desist from offending if they manage to acquire a sense of agency and control over their lives and a more positive outlook on their future prospects [56]. In this context, attributing negative life events to internal and uncontrollable characteristics (e.g. “That’s just the type of person I am”, [24] p187) versus controllable and specific (e.g. behavior), may not engender expectations of change in active offenders. This may have important implications for understanding the process of change in offenders and the potential role of attributional reframing. Maruna suggests that adapting depressogenic attributional thinking patterns may be relevant not only to recovery from depression, but also to the process of desistance from crime in offenders.

### Strengths and limitations

There are two strengths worth noting in the present study. The first is the methodological scope and the theory driven hypotheses. There have been a number of contentious issues raised throughout the evolution of RLHM. The present study sought to address some of these issues that have received relatively little attention in the literature. For example, the role of uncontrollability, the consideration of attributions for positive events, and the specificity of the model to depression while controlling for anxiety. Our findings have a number of potentially important implications for theory and practice. The second strength of this study is the research setting and the sample drawn from a clinically relevant population. Many studies on attributional style and depression, including some of the seminal papers from which the theory has developed, have involved student samples. The continued use of such a specific population subset undoubtedly circumscribes the extent to which findings can be generalized to more clinically relevant samples. The association between attributional style and depression has rarely been investigated in prison samples. This is surprising given the uncontrollable and aversive nature of the prison environment and given findings that individual cognitive differences are relevant to psychological well-being in prisoners. The results from this study suggest that attributional styles may represent important individual cognitive differences that underpin vulnerability to depression in prisoners.

A number of methodological limitations are noteworthy in the present study. Firstly, we only included male prisoners. This may affect the extent to which our findings generalize to the female prison population, who also represent a vulnerable group. Second, we did not examine the diathesis-stress interaction proposed by RLHM. Results from other studies employing a longitudinal design have shown that the cognitive diathesis (i.e. depressogenic attributional style) interacts with the stress (i.e. negative event) to predict changes in depression over time. The cross-sectional design employed herein did not facilitate the assessment of the diathesis-stress interaction. In addition, a negative event was neither operationalized nor measured in the present study. Recent research suggests that the perceived importance of the negative event to the individual is relevant [22]. Perhaps future research could test the diathesis-stress interaction in a longitudinal design [28] with remand prisoners, who are a particularly vulnerable group. For example, at time 1 before trial participants would complete measures of depression, general attributional style, and causal attributions specific to their current circumstances. At time 2, following trial and sentencing, participants who return to prison would be asked to again complete a measure of depression and a measure of their perceptions of the ‘negative event’. This design would facilitate an evaluation of the diathesis-stress interaction and whether it contributes significantly to depression scores at time 2, when controlling for depression scores at time 1. By including a control sample of sentenced prisoners, the study could also clarify whether attributional style contributes to the increasing levels of depression and psychopathology evident in remand prisoners.

The significance of attributional style, as a limited but nevertheless valuable cognitive model of depression should not be overstated. While attributional style accounts for a significant amount of variance in depression in this study, there are other relevant factors that have not been measured in this sample and other relevant theories about the development of depression in prisoners. Depression itself is a complex disorder involving biological states, psychological functioning, and social relationships. Therefore, a comprehensive theory of depression should incorporate biopsychosocial factors and offer explanations of interacting processes and functions. The results reported herein suggest that attributional style may be an important cognitive variable associated with depressive symptoms in prisoners, and hence, that attributional change could help increase resilience. This does not imply that an attributional model offers a sufficient or comprehensive account of depression in prisoners.

## Conclusion

The prevalence of mental health disorders and severity of mental health symptoms in prisoners is indicative of the vulnerability of this population. In general, the mental health needs of this population are poorly appreciated and insufficiently met. There have been increasing numbers detained in prisons over the years, and with the associated increasing pressure on the system to meet the basic needs of prisoners, it is important that we do not neglect prisoners’ psychological well-being. This study emphasizes the importance of developing theory driven psychological models to improve our understanding of mental health problems in prisoners. The findings suggest that an attributional model of depression may be relevant to this population and could provide a valid insight into the development and treatment of depressive symptoms in prisoners. The literature suggests that an enhancing attributional style may be important to recovery from depression. In addition, it has also been associated with desistance from crime in former offenders. In this respect, furthering our knowledge of depressogenic and enhancing attributional styles may be relevant not only for understanding and treating depression in prisoners but also for understanding and facilitating change in cognitions that maintain criminality. This has positive implications for the use of attributional training and reframing with prisoners as part of the ongoing rehabilitative process.

## Supporting information

S1 Dataset Attributional style and depression raw data. (ASPX)

S1 Table Correlation matrix of the predictor and criterion variables. (DOCX)
