# Supplementary material for: Attributional style and depressive symptoms in a male prison sample
Source: PLoS One. 2018 Feb 14;13(2):e0190394. doi: 10.1371/journal.pone.0190394 (PMC5812561; doi:10.1371/journal.pone.0190394)
Supplement: S1 Table — (DOCX) [file pone.0190394.s002.docx]

**Supporting information**

S1 Table. Correlation matrix of the predictor and criterion variables

|  |  | *BDI-II* | *BAI* | *Int Neg* | *St Neg* | *Glo Neg* | *Unctrl Neg* | *Int Pos* | *St Pos* | *Glo Pos* | *Ctrl Pos* |
| --- | --- | --- | --- | --- | --- | --- | --- | --- | --- | --- | --- |
| **BDI-II** | Correlation  Sig 2-tailed | 1.000 |  |  |  |  |  |  |  |  |  |
| **BAI** | Correlation  Sig 2-tailed | .550**  .000 | 1.000 |  |  |  |  |  |  |  |  |
| **Internal Negative** | Correlation  Sig 2-tailed | .227**  .002 | .218**  .003 | 1.000 |  |  |  |  |  |  |  |
| **Stable Negative** | Correlation  Sig 2-tailed | .060  .421 | .015  .842 | .165*  .026 | 1.000 |  |  |  |  |  |  |
| **Global Negative** | Correlation  Sig 2-tailed | .236**  .001 | .180*  .015 | .228**  .002 | .337**  .000 | 1.000 |  |  |  |  |  |
| **Uncontrol Negative** | Correlation  Sig 2-tailed | .077  .296 | .028  .700 | -.083  .258 | .388**  .000 | .184*  .012 | 1.000 |  |  |  |  |
| **Internal Positive** | Correlation  Sig 2-tailed | -.218**  .003 | -.145  .052 | .121  .103 | .054  .466 | -.170*  .021 | -.039  .597 | 1.000 |  |  |  |
| **Stable Positive** | Correlation  Sig 2-tailed | -.115  .124 | -.139  .062 | .103  .163 | .066  .372 | -.056  .448 | .007  .927 | .297**  .000 | 1.000 |  |  |
| **Global Positive** | Correlation  Sig 2-tailed | .082  .269 | .028  .708 | .119  .106 | .028  .707 | .150*  .041 | -.098  .179 | .196**  .008 | .134  .069 | 1.000 |  |
| **Control Positive** | Correlation  Sig 2-tailed | -.150*  .042 | -.178*  .016 | .013  .858 | -.065  .379 | -.175*  .017 | -.207**  .005 | .210**  .004 | .192**  .009 | .070  .338 | 1.000 |

* Correlation is significant at the 0.05 level (2-tailed)

** Correlation is significant at the 0.01 level (2-tailed)
